# *Anopheles maculipennis *complex-responsible for the re-emergence of malaria in Romania?

**DOI:** 10.1186/1756-3305-7-S1-P15

**Published:** 2014-04-01

**Authors:** ML Ivănescu, D Acatrinei, L Miron

**Affiliations:** 1 Department of Clinics, Faculty of Veterinary Medicine, "Ion Ionescu de la Brad" University of Agricultural Sciences and Veterinary Medicine, Iasi, Romania

## 

Between 2010 and 2012, in Iaûi City of Romania 5 species belonging to the complex *Anopheles maculipennis *were identified by PCR: *A. melanoon*, *A. labranchiae, A.atroparvus, A. messeae*and *A. maculipennis sp*. The last three species were involved in the transmission of malaria in Romania between 1892 and 1961. The species *A. labranchiae *was signalled for the first time in Romania, being considered as the main vector of malaria in Europe. The identified samples of *Anopheles labranchiae *were two stage IV larvae, concluding with the adaptation of the species to the climate of Iaûi City. Using a new mathematical model realized and implemented by ourselves, based on the construction of a function of interpolation of Lagrange polynomial type, we realized an extrapolation of the evolution of temperatures for the year 2030, demonstrating the existence of favourable conditions of development of both the vector and the malaria parasite. Therefore, it was a temperature raise by 0.72°C in the whole country, as compared to the period of malaria eradication in Romania, and the extrapolation of the evolution of temperatures in the year 2030 showed a raise by 0.8°C, result which coincides with the prognosis made by prestigious research institutes: NIES, CSIRO, HCCPR, MPIM and NCAR, which show a raise by 0.8-1.7° in 2030. In Iaûi City, the prognosis made for 2030 shows an average of 24°C for this year in the spring-summer months, temperature which is ideal for the development of the life cycle of mosquitoes, but also for the development of the parasite inside the vector.

Taking into account the fact that Iasi, as a cultural city, hosts many foreign students who are carriers of *Plasmodium*, coming from malaria endemic areas, and the immigration of the population for work in countries of the African continent, the human reservoir of *Plasmodium *will be permanently maintained. Correlating the three factors implicated in the transmission of malaria: favourable environmental conditions, presence of the vector and of the human reservoir, we could highlight the growing risk of the re-emergence of malaria in Romania.

